# The feasibility of evaluating radiation dose to the heart by integrating kilovoltage-cone beam computed tomography in stereotactic body radiotherapy of early non-small-cell lung cancer patients

**DOI:** 10.1186/1748-717X-8-295

**Published:** 2013-12-26

**Authors:** Chengxin Liu, Guanzhong Gong, Chen Guo, Tonghai Liu, Jie Lu, Hong Zhao, Wei Dong, Yong Yin

**Affiliations:** 1 Department of Radiation Oncology, Shandong’s Key Laboratory of Radiation Oncology, Shandong Cancer Hospital, School of Medicine and Life Sciences, University of Jinan and Shandong Academy of Medical Sciences, Jinan city, Shandong Province, China; 2 Department of Oncology, Binzhou Medical University Hospital, Binzhou city, Shandong Province, China; 3 JiNan Central Hospital, JiNan city, Shandong Province, China; 4 Department of Radiation Oncology, Shandong Cancer Hospital, JiYan Road 440, Jinan 250117, China

**Keywords:** Non-small-cell lung cancer, Stereotactic body radiotherapy, Kilovoltage-cone beam computed tomography, Heart

## Abstract

**Background:**

To investigate the feasibility of contouring the planning risk organ volume (PRV) for the heart, and to determine the probability of evaluating radiation dose to the heart using kilovoltage-cone beam computed tomography (kV-CBCT) in early-stage non-small-cell lung cancer (NSCLC) patients, who received stereotactic body radiotherapy (SBRT).

**Materials and methods:**

Seventeen NSCLC patients who received SBRT (5Gy/f × 10f dose) were enrolled and subjected to CBCT and CT imaging analyses to plan treatment. Sequential planning CBCT images of individual patient’s hearts were analyzed for reproducibility of heart contouring and volume. Comparative analyses were made between the planning CT- and CBCT-detected heart margins and dose-volume indices for treatment.

**Results:**

The heart volume from planning CT images was significantly smaller than that from CBCT scans (*p* < 0.05), and the volumes based on the different series of CBCT images were similar (*p* > 0.05).The overlap of the heart region on the same anatomical section between the first series of CBCT scans and other scans reached 0.985 ± 0.020 without statistically significant differences (*p* > 0.05). The mean margins of the heart from planning CT and CBCT scans were 10.5 ± 2.8 mm in the left direction, 5.9 ± 2.8 mm in the right direction, 2.2 ± 1.6 mm in the direction of the head, 3.3 ± 2.2 mm in the direction of the foot, 6.7 ± 1.1 mm in the anterior direction, and 4.5 mm ± 2.5 mm in the posterior direction. All relative and absolute dose-volume indices obtained from CBCT images were significantly larger than those from planning CT scans (*p* < 0.05), with the exception of the volume in the 5Gy region.

**Conclusion:**

The PRV of heart contouring based on kV-CBCT is feasible with good reproducibility. More accurate and objective dose-volume indices may be obtained for NSCLC patients by using kV-CBCT, instead of CT, to plan SBRT.

## Introduction

The traditional treatment for patients with early stage (I/II) non-small cell lung carcinoma (NSCLC) is surgery [1]. Unfortunately, several counterindications to surgery exist, such as severe comorbidity, chronic obstructive pulmonary disease (COPD) and/or chronic heart disease [2-4]. Treatments based upon radiation technology have proven useful for NSCLC patients who are not surgical candidates; in particular, stereotactic body radiation therapy (SBRT) has emerged as a safe alternative [5,6].

Studies have suggested that the survival rate for SBRT may be comparable to that for surgery [7]. Moreover, SBRT was shown to provide better clinical outcomes than the conventional fractionated radiotherapy (RT) [8]. Unfortunately, SBRT carries a risk of cardiac toxicity, which reduces its benefit on overall survival [9,10].

Specific cases of adjuvant RT-induced heart disease offsetting improvements in cancer-specific survival have been reported [11]; for example, breast cancer radiotherapy was shown to increase the risk of developing ischemic heart disease, pericarditis, and valvular disease [12]. As a result, radiation oncologists seek to determine the smallest cardiac dose that will provide a particular patient with the maximum efficacy and safety in treating early-stage NSCLC.

SBRT usually involves use of kilovoltage-cone beam computed tomography (kV-CBCT) [13,14] for treatment set-up verification, and CBCT has high spatial resolution and excellent soft tissue imaging capability; therefore, we hypothesized that CBCT may be useful for delineating hearts for the purpose of radiation dose assessment. In addition, the time for CBCT acquisition is 75–90 seconds, which can encompass several respiratory heart motions and cardiac cycles [15]. The purpose of this study was to use CBCT to research the reproducibility of the heart’s planning risk volume (PRV) in early-stage NSCLC patients. In addition, we determined PRV margins [16] for the heart in these patients and estimated the related cardiac dosimetric parameters to perform a comparative analysis with parameters measured by CT scanning.

## Materials and methods

### Patient selection and simulation

Seventeen NSCLC patients who were scheduled to receive SBRT (dose of 5Gy/f × 10f) were enrolled in the study between November 2010 and December 2012. All tumors were histologically staged using the International Association for the Study of Lung Cancer (IASLC) staging system and were determined to be stage I or II. Patient ages ranged between 47 and 76 years, with a median age of 61 years. The patient population included 9 males and 8 females. The pretreatment computed tomography (CT) scanning was carried out with the patient immobilized by evacuated vacuum-bag. CT images (3 mm slice thickness) were transferred to an Eclipse Radiotherapy Treatment Planning System (TPS, version 8.6; Varian Medical Systems, Palo Alto, CA, USA) for contouring and planning, according to ICRU recommendations.

The hospital’s local Institutional Review Board approved the study, and each patient provided informed consent.

### CBCT image acquisition

CBCT images were acquired before each RT session in the treatment room using a scanner attached to the gantry of the Trilogy Linear Accelerator (Varian Medical Systems). Since each RT session consisted of 10 fractions, ten series of CBCT images were acquired to verify set-up accuracy. The CBCT acquisition time was 75 – 90 s at 120 kVp, with various exposures ranging from 0.16 to 0.64 mAs per frame.

### Treatment set-up

The discrepancy between planned and actual tumor position was automatically evaluated using the software accompanying the Trilogy Linear Accelerator. When the quality of a known parameter (such as boney landmarks in the chest) was ambiguous, manual input was performed to facilitate fine adjustment of the standards.

### Contouring the heart on CBCT and CT images

Delineation of the heart was performed using the same display level and window as on the planning CT. Ten seasoned radiation oncologists were involved in contouring the heart ten times for every patient on the CBCT image and one seasoned radiotherapist was asked to contour the heart one time on the CT image, according to a uniform standard. The contouring criteria were as follows: 1) based on anatomical landmarks, the boundary between the cardiac posterior and the esophagus was profiled according to the thickness of the esophagus that filled with gas; 2) the boundaries between the cardiac anterior edge, and the sternum and walls of the chest were distinguished by their continuities of original contours, as well as the CT value; 3) the boundary between the cardiac superior edge and aorta started from the bifurcation layer of the pulmonary artery.

To determine the boundary between the lower edge of the heart and liver, the dropping method of CT value was used. Briefly, we first measured the CT values of several spots at the junction between the liver and heart (spots were chosen based on the investigator’s experience with locating the edge of upper heart and the edge of the junctions between the lower liver and heart). The largest CT value of the several spots in each region was selected and used to connect all the spots to generate a profile of the border of the heart and liver.

### Calculating heart volume, heart overlap, mean heart margins, and dose-volume indices from planning CT and CBCT

The heart volumes from both CBCT and CT images were determined using the Eclipse TPS. Overlapping regions of a heart on the same anatomical section from the series of CBCT imaging were analyzed using MATLAB 2012a software. The mean heart margins from planning CT and CBCT images were obtained after rectification of set-up errors. Dose-volume indices were matched by two-way scanning of the dose volume histogram (DVH). Relative dose-volume indices (V_xGy-R_) were: V_5Gy-R_^,^ V_10Gy-R_, V_15Gy-R_, V_20Gy-R_, V_25Gy-R_, V_30Gy-R,_ and V_35Gy-R_. Absolute dose-volume indices (V_xGy-A_) were: V_5Gy-A_, V_10Gy-A_, V_15Gy-A_, V_20Gy-A_, V_25Gy-A_, V_30Gy-A_, and V_35Gy-A_. Dose indices for fixed heart volume were: D_50cm_^3^, D_100cm_^3^, D_150cm_^3^, D_200cm_^3^, D_250cm_^3^, and D_mean_ (mean dose), D_max_ (exposure dose per 1 cm^3^).

### Statistical analysis

Data are shown as the mean ± standard error of the mean (SEM). Analyses were performed using the Statistical Package for Social Sciences, version 16.0 (SPSS, Chicago, IL, USA). *P*-values less than 0.05 were considered statistically significant.

## Results

### Heart volume

**Figure 1:**
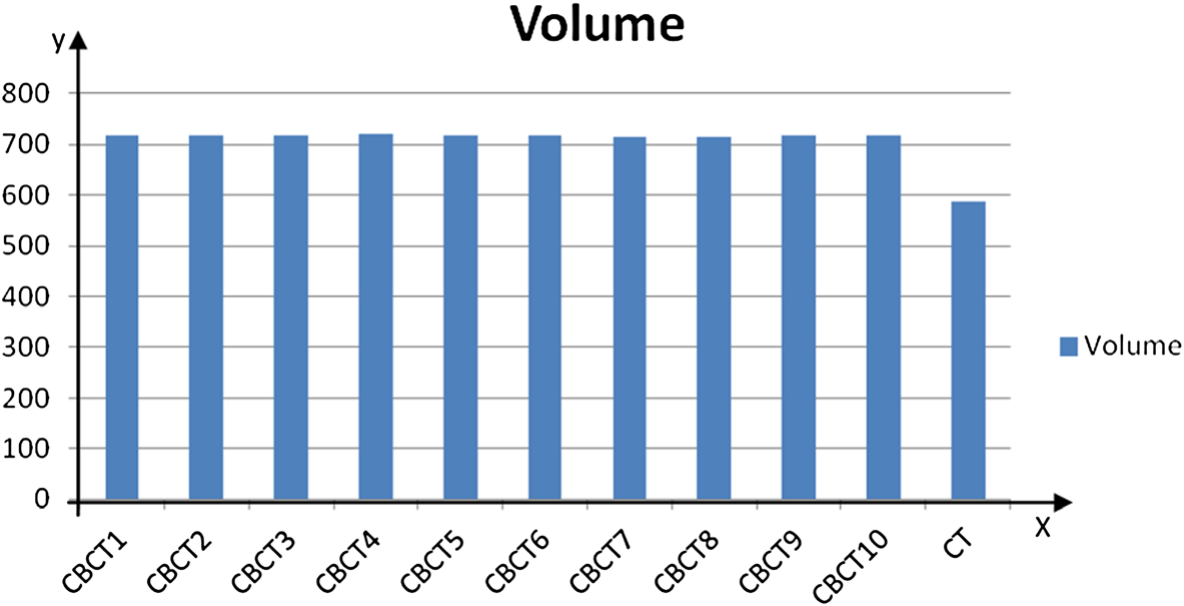
**Mean heart volume of CT and CBCT images.** The volume data of CBCT1 was obtained by averaging heart volumes of the first CBCT images from 17 patients. All other data were obtained similarly.

Figure 1 summarizes the reproducibility of heart volume obtained from different CBCT scans, as well as a comparison of heart volumes between CBCT and CT images. Heart volumes for each patient were largely similar among CBCT images (*F* = 1.00, *p* = 0.44), but were significantly larger than those from planning CT scans (717 ± 100 cm^3^ vs. 588 ± 90 cm^3^; *t* = 4.63, *p* = 0.001).

### Heart reproducibility from a series of different CBCT images

**Figure 2:**
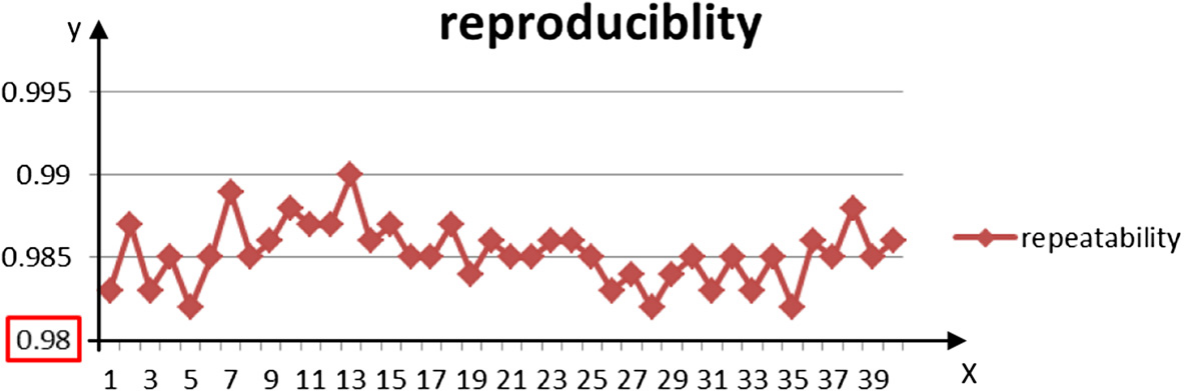
**Heart reproducibility in 40 series.** The fifth layer shows the worst reproducibility, which reaches more than 0.98. The coinciding heart regions on the same anatomical section between the first series of CBCT images and other images reached up to 0.985 ± 0.020 from the first to the 40th section.

**Figure 3:**
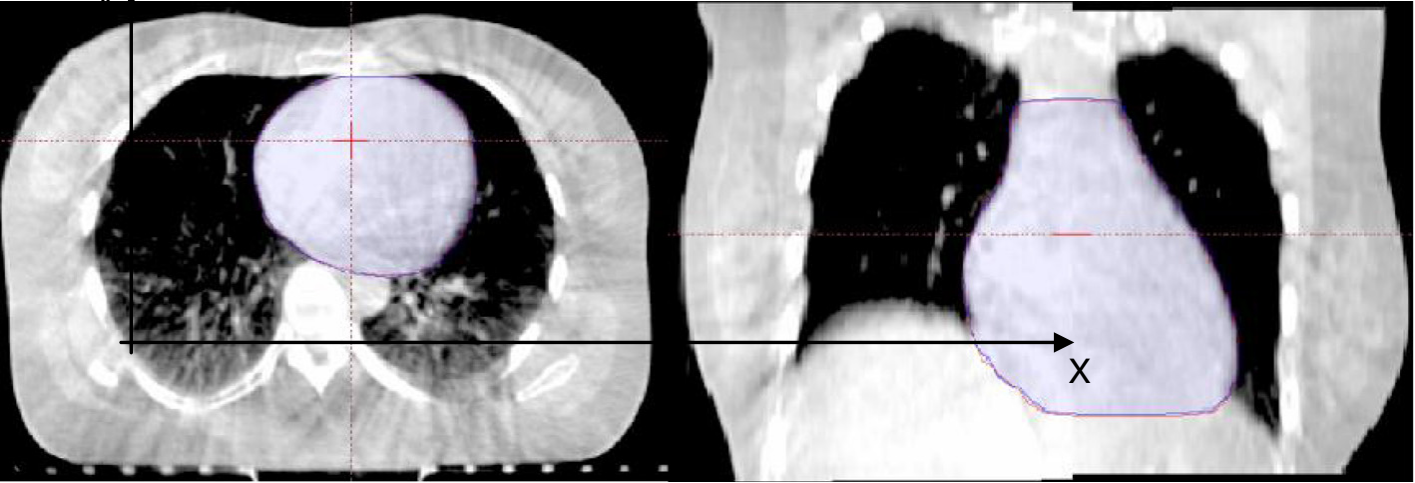
**Reproducibility of CBCT images.** CBCT images of the heart from one representative patient show overlap of the anatomical region. The reproducibility of the heart between the first CBCT (red line) and other images (blue line) are shown on the sectional and coronal planes.

The reproducibility of heart outlines from different series of CBCT images of the same anatomical section (about 40 sections from the collective RT sessions received per patient) reached up to 0.985 ± 0.020 (Figure 2), and the margins of error were not significantly different between each set of images (*p* > 0.05) (Figure 3).

### External margins in different axial directions

**Figure 4:**
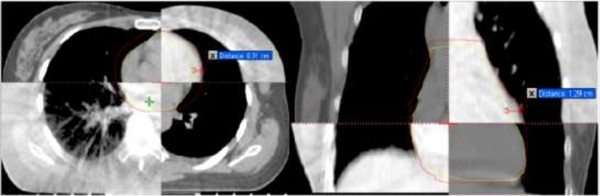
**External margins in different axial directions.** The margin on the sectional direction is 0.91 cm and 1.29 cm on the coronal plane. Similar margins were obtained for all other patient presented.

The margins were measured in every layer after the registration. The maximum distance was given in six different axial directions in an attempt to obtain the most protective outcomes for the heart. The mean margins in different axial directions from the heart on planning CT and CBCT images (pooled data values) were 10.5 ± 2.8 mm in the left direction, 5.9 ± 2.8 mm in the right direction, 2.2 ± 1.6 mm in the direction of the head, 3.3 ± 2.2 mm in the direction of the foot, 6.7 ± 1.1 mm in the anterior direction, and 4.5 mm ± 2.5 mm in the posterior direction (Figure 4).

### Dose-volume indices

**Figure 5:**
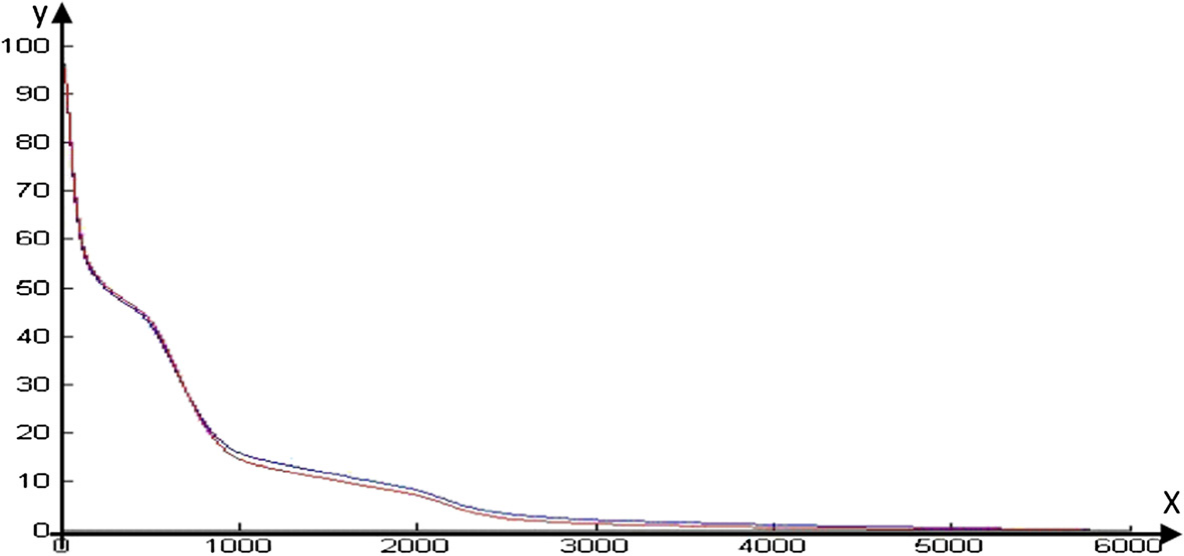
**Mean heart DVH of CT and CBCT images.** Relative dose-volume indices were calculated through the DVH and caves (red line for CT and blue line for CBCT) were obtained using MATLAB 2012a. The two caves are separated.

Relative and absolute dose-volume indices from CBCT images were significantly larger than those from CT images, with the exception of V_5Gy-R_ (*p =* 0.17). In addition, the doses in the fixed heart volumes for CBCT were significantly larger than those for CT (*p* < 0.05), as shown in Table 1 and Figure 5.

**Table 1:** Dose-volume indices between CBCT and CT

**Parameter**	**CBCT, ± SEM**	**CT, ± SEM**	***t***	***P***
V_5Gy-R_^a^	47.7 ± 16.4%	49.0 ± 16.0%	1.5	0.17
V_10Gy-R_	18.9 ± 11.7%	17.4 ± 11.8%	2.3	0.03
V_15Gy-R_	14.4 ± 10.2%	12.8 ± 10.4%	2.3	0.02
V_20Gy-R_	10.5 ± 9.0%	9.0 ± 8.9%	2.3	0.04
V_25Gy-R_	6.6 ± 2.8%	4.5 ± 2.3%	3.6	0.01
V_30Gy-R_	4.2 ± 2.2%	2.5 ± 1.6%	3.7	0.01
V_35Gy-R_	3.2 ± 1.8%	1.7 ± 1.3%	3.9	0.01
V_5Gy-A_^b^	333.0 ± 78.4 _cm_^3^	282.0 ± 61.7 _cm_^3^	7.9	0.0001
V_10Gy-A_	133.0 ± 68.6 _cm_^3^	101.0 ± 59.0 _cm_^3^	6.8	0.0001
V_15Gy-A_	102.0 ± 62.6 _cm_^3^	74.6 ± 53.7 _cm_^3^	6.2	0.0001
V_20Gy-A_	74.0 ± 55.8 _cm_^3^	52.5 ± 46.4 _cm_^3^	4.8	0.0001
V_25Gy-A_	46.0 ± 16.9 _cm_^3^	27.0 ± 12.0 _cm_^3^	6.2	0.0001
V_30Gy-A_	30.0 ± 14.4 _cm_^3^	15.0 ± 8.7 _cm_^3^	5.5	0.0001
V_35Gy-A_	24.0 ± 12.0 _cm_^3^	10.0 ± 7.0 _cm_^3^	5.5	0.0001
D_50cm_^3^^c^	19.9 ± 7.7 _Gy_	17.1 ± 6.3 _Gy_	4.5	0.0001
D_100cm_^3^	15.3 ± 6.2 _Gy_	12.8 ± 5.7 _Gy_	3.6	0.003
D_150cm_^3^	11.8 ± 5.5 _Gy_	9.2 ± 3.4 _Gy_	3.6	0.003
D_200cm_^3^	8.2 ± 2.0 _Gy_	6.8 ± 1.5 _Gy_	6.2	0.0001
D_250cm_^3^	6.4 ± 1.7 _Gy_	5.2 ± 2.0 _Gy_	6.5	0.0001
D_mean_	7.0 ± 2.8 _Gy_	6.5 ± 2.4 _Gy_	3.0	0.009
D_max_	45.7 ± 13.1 _Gy_	45.1 ± 13.5 _Gy_	2.9	0.009

a). V_5Gy-R_ means the heart relative volume in the 5Gy region, V_10Gy-R_, V_15Gy-R_, V_20Gy-R_, V_25Gy-R_, V_30Gy-R,_ and V_35Gy-R_ are of the same mean;

b). V_5Gy-A_ means the heart absolute volume in the 5Gy region, V_10Gy-A_, V_15Gy-A_, V_20Gy-A_, V_25Gy-A_, V_30Gy-A_, and V_35Gy-A_ are of the same mean;

c). D_50cm_^3^ means the exposure dose in the 50 cm^3^, D_100cm_^3^, D_150cm_^3^, D_200cm_^3^, and D_250cm_^3^ are of the same mean.

## Discussion

Over the last half century, RT has evolved to become one of the cornerstones of treatment for various types of thoracic cancers. However, radiation-related heart disease (RRHD) resulting from radiation therapy for the treatment of certain malignancies is becoming an increasing concern for patients and clinicians alike [10,17]. The spectrum of pathology affecting the heart spans from acute to chronic, and can affect almost all facets of the heart [18]. Radiation damage to the heart can occur several months to years after treatment, and frequently culminates in congestive heart failure, ischemia, coronary artery disease, or myocardial infarction [19].

The long-term effects of RT on the cardiovascular systems remain an important research issue and studies have shown that relative risks increase with higher radiotherapy doses, younger age at irradiation, and the presence of conventional risk factors [17,20]. Even at lower radiation doses, excess risk of cardiovascular disease occurs a long time after exposure, as demonstrated in Japanese atomic bomb survivors [21]. This makes the biologically effective dose more complex and important, both to the targets and to the heart, due to the increase in dose per fraction when early-stage lung cancer cases are treated with SBRT [8]. Hence it is of great importance to accurately assess the radiation dose delivered to the heart.

Currently, the risk of cardiac events correlates with dose-volume predictors, which are calculated based on planning CT images; however, these images do not reflect the real shape and volume of the heart, as they only show the location and shape of the heart in each layer of the CT image at a specific moment in time under the influence of breathing and heartbeat. Therefore, great differences regarding the estimations of dose-volume predictors for RRHD have been obtained among the various institutional studies performed to date.

Four-dimensional computed tomography (4D-CT), combined with a respiratory tracking system and volume scanning to obtain dynamic time-relevant images, can provide an accurate measure of the breathing-induced anatomical motion of the patient [22], and can also perfectly eliminate the influence of respiratory movements in clinical applications [23]. Thus, 4D-CT is now accepted as a standard tool for simulation and treatment planning in lung SBRT [24,25]. Compared to the length of respiratory movements, however, the heartbeat is so short that 4D-CT cannot accurately reflect the heart’s condition. The best way to evaluate irradiation dose of the heart is application of the electrocardiography gating dynamic magnetic resonance imaging (MRI) analysis. However, deformable image registration is needed for different MRI phase images and the registration between MRI and planning CT. The accumulation among different phase dose are also required. Furthermore, these technologies have not yet formed a mature evaluation process.

It is important to note that the electrocardiographically-gated method cannot be used to simulate CT in determining the heart PRV. Since SRBT patients generally require a high irradiation field dose and long irradiation time, it is difficult to apply ABC in these patients. The high dose rate of the SBRT is anticipated to solve this problem. Truebeam is able to reach a dose rate of 2400 MU/min, but its reproducibility is weak. At present, only a few reports about the efficacy and safety of SBRT in combination with ABC exist.

CBCT imaging, which encompasses several respiratory and cardiac movement cycles, represents an effective means of graphing the heart PRV to plan for SBRT. Using a rotating kilovoltage x-ray source and a flat panel detector has improved the CBCT technique [15,26]. In addition, not only can CBCT enhance the precision of radiotherapy for target volumes, but it can also reduce the radiation volume and dose for organs at risk [27]. As reported by Nakamura *et al.*, free-breathing CBCT acts as a slow CT scan that contains target motion [28]. The kV-CBCT scan period is relatively long (75 – 90 s), and includes 12–20 respiratory cycles and more than 100 cardiac cycles [13]. This feature allows the avoidance of the influence of respiratory and cardiac movements, resulting in a more accurate representation of the heart’s motion. The CBCT scan also includes the movements of many organs, so it has unique advantages for researching the PRV of the heart. Topolnjak *et al.* applied CBCT to studying the PRV of the heart in the treatment of breast cancer, and presented specific external margins [29]; however, their research only considered the breathing cycles and failed to include the influence of heartbeats.

In the current study, the heart was contoured under a unified standard with the application of CBCT and included breathing and heartbeat, which are the two main factors that influenced the heart location in the thoracic cavity. In addition, the external margins were calculated by comparison with planning CT images, which could better reflect the actual location of the heart. Finally, all relative and absolute dose-volume indices were compared in this study because there is not a clear relationship between dose-volume indices and RRHD [9]. The poor image quality and motion artifact of the CBCT could have an impact on contouring the heart. This impact could be minimized by the following methods:

1. The kV-CBCT scan period was so long that the image change caused by motion might be blurred. Therefore, the image deflection and torsion would be reduced, while the setup error was corrected using the chest bony landmarks as the judgment standard.

2. After the registration of CBCT image to the planning CT images, delineation of the heart would be performed in the same window as the CT.

3. When the heart was closed to the surrounding tissues, the dropping method of CT value was used to distinguish the heart from the surrounding tissues.

4. In this paper, the outline of the heart was contoured and fine structure, such as the left anterior descending coronary artery, was not delineated. Therefore, the CBCT image resolution was sufficient to contour the heart.

There was 95% reproducibility of heart images based on 10 CBCT scans, which manifested as overlapping of the heart’s region on the same anatomical section between the first series of CBCT scans; the error of the heart volume was within 5%. For this reason, the heart graph and volume from the first CBCT scan were enough to replace the others. In this way, the time of heart contouring based on CBCT can be reduced, which can also relieve the risk to the heart from the CBCT, particularly for patients who have a history of heart disease.

By comparing the first series of heart CBCT and CT images, we found that the heart boundary from CBCT images was larger than that from CT images, and the difference was even greater in the left anterior lower area of heart where the heart movement was most obvious with the largest distance of 13 mm. Throughout the study, we concluded that it was necessary to conduct CBCT scans in patients who might suffer RRHD when receiving SBRT for treatment of NSCLC.

Although heart ranges based on CBCT images were larger than those based on CT scans, their boundaries were much closer to the tumor. Therefore, the relative and absolute dose-volume indices correspondingly became larger. With the additional comparison of dosimetric parameters in DVHs, all indices for heart doses based on CBCT scans were larger than those based on CT scans, with the exception of V_5Gy-R_. Therefore, the application of kV-CBCT for the estimation of radiation doses to the heart is a better method for protecting the heart in early stage NSCLC patients receiving SBRT treatment. Furthermore, for patients with tumors near the heart or who are inoperable due to heart disease, it is extremely important to accurately estimate the radiation dosage to the heart and to forecast cardiac injury with the application of CBCT. This technique provides a more objective, scientific and general method of heart dose estimation in the application of SBRT for treating early-stage NSCLC. Our future studies will include analyses of the relevance between CBCT dose-volume indices and RRHD.

In conclusion, determining the PRV of heart contouring based on kV-CBCT is feasible and shows good reproducibility. More accurate and objective dose-volume indices may be obtained from applying kV-CBCT in the SBRT of early-stage NSCLC patients.

## Abbreviations

PRV: Planning risk organ volume; kV-CBCT: Kilovoltage-cone beam computed tomography; NSCLC: Non-small-cell lung cancer; SBRT: Stereotactic body radiotherapy; COPD: Chronic obstructive pulmonary disease; RT: Radiotherapy; DVH: Dose volume histogram; SEM: Standard error of the mean; RRHD: Radiation-related heart disease; CHF: Congestive heart failure; CAD: Coronary artery disease; MI: Myocardial infarction; BED: Biologically effective dose; 4D-CT: Four-dimensional computed tomography.

## Competing interests

The authors declare that they have no competing interests.

## Authors’ contributions

CX L carried out outlining the heart, participated in collecting the CBCT images and drafted the manuscript. WD and HZ carried out the MATLAB 2012a software. TH L helped outline the heart. JL helped collect the CBCT images. GZ G and CG participated in the design of the study and performed the statistical analysis. YY conceived of the study, and participated in its design and coordination and helped to draft the manuscript. All authors read and approved the final manuscript.
